# Acknowledgement to Reviewers of *Nutrients* in 2018

**DOI:** 10.3390/nu11010145

**Published:** 2019-01-11

**Authors:** Nutrients Editorial Office

**Affiliations:** MDPI, St. Alban-Anlage 66, 4052 Basel, Switzerland

Rigorous peer-review is the corner-stone of high-quality academic publishing. The editorial team greatly appreciates the reviewers who contributed their knowledge and expertise to the journal’s editorial process over the past 12 months. In 2018, a total of 2031 papers were published in the journal, with a median time to first decision of 14.00 days and a median time to publication of 37.00 days. The editors would like to express their sincere gratitude to the following reviewers for their cooperation and dedication in 2018:

A. Frame, LeighLiang, YanA. Frassetto, LyndaLiang, ZhibinAaron, Jean E.Liao, Jyh-feiAbbas, AzharLiberale, LucaAbbeddou, SouheilaLichtenberg, DovAbbott, David H.Liddell, Jeffrey R.Abenavoli, LudovicoLiem, GieAbeywardana, TharindumalaLightfoot, AdamAbles, GeneLila, Mary AnnAbraham, Bincy P.Lillioja, StephenAbraham, Elizheeba ChristieLim, HyunjungAbrams, Barbara FLim, Ji-HongAbrams, StephenLim, KarenAbreu, SandraLim, Karen H. C.Abu, Brenda Ariba ZarhariLim, Wai H.Abuelo, ÁngelLin, Chih-LiAceti, AriannaLin, Chung-Tung J.Adams, Dawn WieseLin, HoAdams, Katherine P.Lin, Hung-YinAdams, SeanLin, Pao-HwaAddo, O. YawLin, Shyh-HsiangAdegoke, Olasunkanmi A. JohnLinder, MariaAdhikari, NeetaLindsay, KarenAdjonu, RandyLintig, JohannesAgarwal, EktaLisciani, SilviaAghajafari, FaribaLittle, JonathanAglago, Elom KouassiviLittlejohns, ThomasAgnew, TamaraLiu, AipingAgostoni, CarloLiu, ChangAgrawal, SwatiLiu, EnjuAguiar, ElroyLiu, Guang-YawAhmad, AamirLiu, HexuanAhmad, ShakilLiu, Hui-MingAhmed, FaruqLiu, JianAhmed, MavraLiu, JianfengAhn, Mok-RyeonLiu, JunAhrens, Richard A.Liu, LingAhuja, SumanLiu, Pei-YangAkombi, Blessing J.Liu, Shing-HwaAlam, Md BadrulLivesey, GeoffreyAlao, John PatrickLlanos Chea, AlejandroAlashi, Adeola MLloyd, Megan LAl-Dujaili, EmadLo Monte, Attilio IgnazioAlexander, YvonneLo Scalzo, RobertoAlexandre-Gouabau, Marie-cécile F.Lo Siou, GeraldineAlexy, UteLobo, GlennAl-Fatimi, MohamedLockridge, OksanaAli, AjmolLocks, LindseyAlique, MatildeLoewendorf, Andrea I.Al-Jawaldeh, AyoubLogue, CaomhanAllegaert, KarelLohner, SzimonettaAllen, LindsayLøken, Elin BAlley, ThomasLoman, BrettAlmeida Palha, JoanaLombardi, KarenAlmoosawi, SuzanaLombó, FelipeAlonso-Magdalena, PalomaLonardo, AmedeoAlsharairi, NaserLonchamp, JulienAltemimi, AmmarLondon, EdraAltosaar, IllimarŁoniewski, IgorAlvares, ThiagoLópez Jornet, PiaAmarasena, NajithLopez Lopez, DanielAmbigapathy, GaneshLópez Sánchez, Guillermo F.Ambroszkiewicz, JadwigaLópez Sobaler, Ana MaríaAmengual, JaumeLópez, Inés VelascoAmigo-Benavent, MiryamLopez, NanetteAmillis, SotirisLópez, VíctorAmir, MohammedLopez-Escamez, Jose A.Amirabdollahian, FarzadLópez-Hernández, ArnoldoAmodio, PieroLópez-López, DanielAnandhan, AnnaduraiLópez-López, JoséAnanieva, ElitsaLoria, AnaliaAndersen, Barbara VadLoser, KarinAndersen, CatherineLosurdo, GiuseppeAndersen, Jens RikardtLouis, JulienAndrade, Jeanette M.Louis, SandrineAndrade, Paula BLourida, IliannaAndreev, KonstantinLove, PennyAnes, ElsaLu, Jeng-WeiAnestis, DougkasLu, JunAnestopoulos, IoannisLu, Kuo-ChengAngadi, SiddharthaLu, Louise WeiweiAngelino, DonatoLu, Mei-ChinAngelone, TommasoLu, Mei-KuangAnifandis, GeorgeLubahn, DennisAnnibale, BrunoLucarini, MassimoAnower-E-Khuda, Md. FerdousLucas, RudolfAnsa, BenjaminLuccia, Aldo DiAnsari, RaisLucey, AliceAnsorena, DianaLudy, Mary-JonAnsquer, Jean ClaudeLuick, BretAntar, AlliLuis Ubago, JoseAnthony, TracyLuís, ÂngeloAnton, StephenLukaski, Henry C.Anton, Stephen D.Luna, AurelioAntonio, Javier Mateu-deLund, Elizabeth K.Antony, JaneLunn, WilliamApekey, TanefaLuo, JinAppleton, KatherineLuo, ZonghuaAragonès, GerardLuthra, PriyaArai, ToshiroLütjohann, DieterArana, AnaLutz, ThomasAraújo, Maria EduardaLuu, Hung NArcher, DavidLux-Battistelli, ChristineArcher, EdwardLynch, Heidi M.Ardévol, AnnaLynch, KrystalArena, SabrinaLyons, GrahamArends, JannLyons, Paul E.Arentson-Lantz, EmilyMa, HangAres, GastonMa, LongArihara, KeizoMa, YiyiArjmandi, BahramMa, YunshengArmah, Seth M.Ma, Zheng FeeiArmand, MartineMaalouf, NaimArnedo, AlbertoMabhala, MzwandileArnoldi, AnnaMachado Jr, Julio CAronsson, Carin AndrénMack, IsabelleArosio, PaoloMacKay, DylanArthur, RhondaMackerras, Dorothy E MArtunc, FerruhMadar, Ahmed A.Asano, ShinichiMadhukar, BurraAsatryan, BabkenMadore, CharlotteAsbell, Penny A.Madzima, Takudzwa A.Ashish, PathakMaestro, MiguelAshorn, UllaMagini, AlessandroAshrafi, Hossein H.Maglio, MariantoniaAshton, JamesMahajan, SahilAshton, LeeMahmud, FaridAslam, MuhammadMahoney, Sara E.Aspatwar, AshokMahony, Timothy JohnAssaf-Balut, CarlaMai, StefaniaAssanelli, DeodatoMai, VolkerAssari, ShervinMaietti, AnnalisaAstbury, NerysMajumder, KaustavAterini, StefanoMajumder, MrinmoyeeAtkinson, FionaMaki, KevinAtkinson, StephanieMalaguarnera, GiuliaAtteritano, MarcoMalin, StevenAttuquayefio, TukiMalissiova, EleniAugustin, LiviaMalkova, DaliaAune, DagfinnMallard, Simonette R.Averbeck, DietrichMallo, FedericoAydemir, TolunayMaloberti, AlessandroAyers, PhilipMalomo, Sunday A.Ayonrinde, OyekoyaMaltais, MathieuAyonrinde, Oyekoya T.Manary, MarkAzab, AbedMancini, AnnamariaAzcona, María CristinaMandala, AshokAziz, Abdul RashidMandalari, GiusyBabraj, John AndreeMandato, ClaudiaBachschmid, Markus MichaelMangels, ReedBackstrand, Jeffrey R.Manjunath, ManuboluBadenhorst, ClaireMannan, HaiderBado, IgorManoli, IriniBae, SoochanManore, MelindaBafor, Enitome E.Manresa, PabloBahnson, EdwardMansour, SamerBai, RenrenMansueto, PasqualeBailey, ReganManthey, John A.Bajpai, RichaManti, SaraBajpai, VivekMantzioris, EvangelineBakht, MartinMantzoukas, SpiridonBakillah, AhmedMantzouridou, FaniBakker, Marije FManuelli, MatteoBakker, StephanManzanera, SilvestreBalantekin, KatherineManzo, CiroBalcerczyk, AnetaMarangoni, FrancaBaldassarre, Maria ElisabettaMarasco, Lisa AnnBallarotto, GiuliaMarchesini, GiulioBally, LiaMarchetti, GiovannaBälter, KatarinaMarek, Ryan J.Bambling, MatthewMares, JulieBandres-Ciga, SaraMaresca, MarcBandyopadhyay, BidhanMaret, WolfgangBanerjee, KalpitaMargerison, ClaireBanerjee, SudipMaria José, Rodriguez LagunasBang, Byoung WookMarian, Mary J.Bañuelos, GaryMarigliano, MarcoBao, Yong-PingMarin, Daniela ElizaBarabas, PeterMarini, ElisabettaBarabasi, Albert-LaszloMarino, JosephBaracos, VickieMariotti, FrançoisBaragetti, AndreaMarkaki, AnastasiaBarak, MiraMarklund, MattiBaran, MarzenaMarletta, LuisaBarbagallo, DavideMarranzano, MarinaBarbaro, BarbaraMarriott, Bernadette P.Barbieri, AntonioMarroqui, LauraBarbone, AlessandroMarshall, AutumnBarca, AmilcareMarshall, Teresa A.Barchetta, IlariaMarteau, PhilippeBärebring, LinneaMårtensson, JohanBarffour, Maxwell A.Martin, CamiliaBarichello, TatianaMartín, FranzBarnard, NealMartin, JillyBarnes, KateMartin, Stephanie L.Barnhill, KellyMartín-Aragón, SagrarioBarniol-Xicota, MartaMartínez Sanz, José MBarrea, LuigiMartinez, HomeroBarreira, João C.M.Martinez, KristinaBarrott, JaredMartínez-Cañamero, MagdalenaBartelt, AlexanderMartínez-Galiano, Juan MiguelBarzilay, Joshua IMartínez-Tomé, MagdalenaBasak, DebasishMartini, ClaudiaBasak, Saroj K.Martini, DanielaBasini, GiuseppinaMartins, NatáliaBassareo, Pier PaoloMaruf, Abdullah AlBasso, KariMaruyama, KeiBasu, ArpitaMarverti, GaetanoBatarseh, YazanMarwarha, GurdeepBattram, Danielle S.Marx, WolfBaum, JamieMarx, WolfgangBaur, Daniel A.Marzocco, StefaniaBavaro, Simona LuciaMasaki, TakayukiBayat, ArezouMasala, CarlaBazzano, AlessandraMascherini, GabrieleBazzucchi, IleniaMaslin, KateBeasley, SheaMaslova, EkaterinaBeberashvili, IliaMason, LauraBeckett, EmmaMassot, MalenBeharry, Kay D.Masulli, MariaBekhit, Alaa El-Din A.Mathews, Anne E.Bellavia, DanieleMatinolli, Hanna-MariaBellisle, FranceMatsubara, KeiichiBello, Nicholas T.Matsuda, ShinjiBenatar, Jocelyne RMatsufuji, HiroshiBenatar, Jocelyne R.Matsumoto, HiroyukiBenedec, DanielaMatsumoto, YasuhikoBenetou, VassilikiMatsunaga, YutakaBenowitz, Larry I.Matteoli, GianlucaBenson, SarahMatthews, EvanBerendsen, AgnesMattner, JochenBerens, Pamela D.Maugeri, AndreaBerg-Beckhoff, GabrieleMavangira, VengaiBergdahl, AndreasMavrommatis, YiannisBerger, GregorMawhinney, ThomasBerger, Mette MMawson, AnthonyBergeron, NathalieMayer-Proschel, MargotBergheim, InaMazur-Bialy, Agnieszka IrenaBernhard, WoflgangMazzocchi, AlessandraBernini, RobertaMc Manus, RossBernstein, JodiMc Naughton, Lars RBernstein, MelissaMcAllum, ErinBerrett, Andrew N.McAuliffe, Fionnuala M.Berry, Ann AndersonMcCabe, Laura R.Berthier, CelineMcCaskill, MichaelBerthoud, Hans-RudolfMcComb, JacalynBertram, Hanne ChristineMcCormack, LaceyBesnier, FlorentMcCormick, RachelBeto, Judith A.McCourt, PeterBettazzi, FrancescaMcCrickerd, KeriBettendorff, LucienMcCully, KilmerBharadwaj, ShishiraMcfarland, Lynne V.Bhat, NehaMcGlory, ChrisBhatia, JatinderMcGrath, RachelBhattacharjee, SonaliMcGregor, Neil R.Bhattacharya, AnannyaMcHill, Andrew WBiagi, FedericoMcInnes, RhonaBiagi, MarcoMcJarrow, PaulBianchi, VittorioMcKay, Naomi J.Bidarimath, MallikarjunMcKenna, Malachi J.Bidault, GuillaumeMcKinley, MichaelBidwell, Amy J.McLachlan, Craig StevenBigornia, Sherman JMcLaren, MargaretBihuniak, JessicaMcLean, Rachael M.Bijak, MichałMcLennan, PeterBillaud, MarieMcMahon, EmmaBinns, ColinMcNeil, JessicaBiondani, GiuliaMcNulty, BreigeBiondi, MarcoMcPherson, Nicole O.Bird, Julia K.Meadus, William JonBirringer, MarcMearns, GaelBiruete, AnnabelMears, Stephen A.Bishop, MonicaMedler, Kathryn F.Bizzaro, DeboraMehla, JogenderBjørke Monsen, Anne-LiseMeier, Kathryn E.Blachier, FrancoisMeier, PaulaBlack, LucindaMeinilä, JelenaBlack, NicolaMeisinger, ChristaBlais, AnneMekary, RaniaBlando, FedericaMekary, Rania A.Blaney, SoniaMelini, FrancescaBlanton, CynthiaMelkani, GirishBlat, SophieMellendick, KevanBlatchford, PaulMelnik, BodoBlekkenhorst, LaurenMemon, Muhammed AshrafBlomstrand, E.Mendes, AnaBlumberg, JeffreyMendes, RosaBo, Cristian DelMéndez, LucíaBoaz, MonaMeneguzzo, FrancescoBobart, LauraMeng, YuBocos, CarlosMeng, Yuan XiangBodily, JasonMenon, KavithaBogdański, PawełMento, CarmelaBøhn, Siv KMera, PaulaBohn, TorstenMercadel, LucileBohrer, Benjamin M.Mercado, Carla IBold, JustineMerino, RamónBolhuis, DieuwerkeMeruvu, SunithaBollag, WendyMerz, BenediktBolland, Mark J.Mesquita, JoãoBondoc, IonelMessaritakis, IppokratisBondonno, CatherineMessini, Christina I.Bondonno, Catherine P.Messner, DonaldBondonno, Nicola P.Mestres, ConxitaBongers, KaleMeyer, Katie A.Bonilla, CarolinaMeyer, RosanBonnefont-Rousselot, DominiqueMeyer, SörenBonomini, FrancescaMeynier, AnneBonos, EleftheriosMeyre, DavidBoquien, Clair YvesMiao, JiBorbély, YvesMiccadei, StefaniaBorchman, DouglasMichaelidou, Alexandra-MariaBorengasser, Sarah J.Michaelsen, KimBorer, Katarina T.Michels, Alexander J.Borrelli, OsvaldoMichos, AthanasiosBorriello, AdrianaMicillo, RaffaellaBosch, ChristineMiele, ClaudiaBosch, JoaquimMiele, Nicoletta AntonellaBosland, Maarten C.Mielgo-Ayudo, JuanBoström, Kristina I.Mietlicki-Baase, ElizabethBotet, FrancescMiguel, MartaBotham, KathleenMikhailov, Theresa ABotturi, AndreaMikhailova, AlexandraBougma, KarimMiki, YasuhiroBourdon, EmmanuelMikkelsen, Bent EgbergBourgeois, DenisMilagro, FerminBoušová, IvaMilan, AmberBowen, DeborahMiletta, MariaBowrey, David J.Millar, MichaelBowtell, JoannaMiller, JacquelineBoysen, ReinhardMiller, MarkBozonet, Stephanie M.Mills, KerryBozzetto, LutgardaMinato, Ken-ichiroBraakhuis, AndreaMintz, YonatanBradshaw, PatrickMinucci, SergioBraesco, VéroniqueMiotto, PaulaBrameld, JohnMirali, MahlaBrandhagen, MartinMiranda, JonatanBrantsæter, Anne LiseMirhosseini, NaghmehBrasó-Maristany, FaraMisciagna, GiovanniBraud, F. DeMishra, AbheepsaBravenboer, NathalieMistura, LorenzaBravo Vásquez, Francisca IsabelMisurcova, LadislavaBrecht, M.Mitchell, CameronBrestoff, Jonathan R.Mitchell, DianeBrewer, GeorgeMiura, KyokoBrewster, Lizzy MMiyata, JunBriassoulis, GeorgeMiyazaki, TeruoBrigelius-Flohe, ReginaMiyazaki, TetsuroBriggs, Marc AMiyoshi, TakekazuBril, FernandoMizuno, Cassia SBrindal, EmilyMizuno, KatsumiBriskey, DavidMizuno, TooruBroadley, MartinM’koma, Amosy E.Broderick, Tom L.Mnif, WissemBrooke, JoanneMobbili, GiovannaBroom, IainMoeser, Adam J.Brottveit, MargitMohamed, Salah A.Brough, LouiseMohd, Farooq ShaikhBrown, AndrewMohn, EmilyBrown, CallieMolendijk, Marc L.Brown, KatieMollen, Kevin P.Brown, Robert J.Moltu, SisselBrownlee, IainMond, JonBrownlee, Iain A.Monda, VincenzoBruneau Jr, MichaelMoner, VerónicaBruun, SigneMonk, JenniferBrzeziński, MichałMonro, John.Buckley, M.Montalcini, TizianaBucolo, ClaudioMonteagudo, AndreaBuczkowski, BartoszMonteleone, IvanBuddington, Randal K.Montesano, DomenicoBudhathoki, SanjeevMontgomery, MagdaleneBudryn, GrażynaMontgomery, McKaleBueno Cavanillas, AuroraMook-Kanamori, D.O.Bührer, ChristophMoon, MinhoBulk, EtmarMoore, AmandaBunik, VictoriaMoore, GeromyBunn, DianeMoores, CarlyBuonocore, DanielaMorabito, RossanaBurd, NicholasMorag, IrisBurden, Richard J.Moraga, GemmaBurgess, JohnMorales Suárez-Varela, María M.Burghardt, KyleMoranta Mesquida, DavidBurgos-Ramos, EmmaMoreau, RegisBurke, LouiseMorelli, SabrinaBurnett, Alissa J.Moreno, Diego A.Burns, DavidMoreno, Juan JoséBurns, Ryan DonaldMoreno-Navarrete, José María MorenoBurrows, TracyMoretti, DiegoBurtea, CarmenMorganti, PierfrancescoBurtey, StéphaneMorgese, Maria GraziaBurton, Elvin ThomaseoMorimura, ShigeruBurton-Freeman, BrittMorino, KatsutaroButel, Marie-JoséMorioka, TomoakiButts, Christine AMorisset, Anne-SophieByrd-Williams, CourtneyMorita, ManabuByrne, RebeccaMörkl, SabrinaCabañero-Martínez, Maria JoseMorozov, VasilyCabanillas, BeatrizMorris, AndrewCádiz-Gurrea, María LuzMorris, HowardCahova, MonikaMorriset, Anne-SophieCai, DeminMorroni, FabianaCaiazzo, ElisabettaMosca, AndreaCairrão, ElisaMosca, LucianaCalamita, GiuseppeMoser, OthmarCaldarelli, StefanoMosoni, LaurentCalder, PhilipMost, JasperCaldwell, Kathleen L.Motyl, KatherineCalejman, Camila MartinezMougiakakou, StavroulaCalle-Pascual, Alfonso L.Mounien, LourdesCallery, Patrick S.Mounier, CatherineCalon, FrédéricMoursi, MouradCamera, DonnyMousa, AyaCamilleri, MichaelMousa, Shaaban A.Camire, MaryMovassagh, HesamCamire, Mary EllenMu, QinghuiCampbell, Fiona M.Mueller, Megan P.Campbell, Grace J.Mukhtar, HasanCampbell, MatthewMulder, MoniqueCamps, GuidoMuldoon, Matthew FrancisCanfora, Emanuel E.Mulla, Christopher MCanini, AntonellaMuller, Dominik N.Cannell, John J.Muller, Manfred JamesCantorna, MargheritaMüller-Alcazar, AnettCao, Jay JMulligan, Angela ACapaldo, BrunellaMulvihill, ErinCapasso, RaffaeleMunblit, DanielCapel, FredericMundel, TobyCapel, FrédéricMuñiz Rodriguez, PilarCapozzi, VittorioMunoz, ColleenCaputi, ValentinaMuñoz-Alférez, M. JoséCarabotti, MariliaMurach, KevinCarapella, LauraMuramoto, KojiCardenas Zuniga, RobertoMurphy, JaneCardenia, VladimiroMurray, KathrynCardinali, AngelaMurray, MargaretCardoso, SusanaMurry, Daryl J.Carella, Angelo MMusch, MarkCarey, AmandaMusher-Eizenman, DaraCarl, DanielMushtaq, SohailCarlberg, CarstenMusso, NataleCarlsen, Monica H.Mustad, Vikkie A.Carluccio, MariaMuthuramu, IlayarajaCarmena, DavidMyers, StephenCarne, AlanMyhre, Jannicke BorchCarnés, JerónimoMyneni, AjayCarocho, MarcioMyracle, AngelaCarpene, ChristianMyszkowska-Ryciak, JoannaCarpenter, GHNa, MuziCarr, AnitraNagai, YoshinoriCarra, Maria ClotildeNagpal, RavinderCarragher, JohnNaik, SurabhiCarrie, GrueterNairz, ManfredCarrillo-Sepulveda, Maria AliciaNakagawa, YoshimiCarson, BrianNakaki, ToshioCaruso, CalogeroNakamaru-Ogiso, EikoCaruso, GianlucaNakano, YuyaCasanovas, CarmenNakhauka, Ekesa BeatriceCascone, SaraNam, Ju-OckCasini, GiovanniNambiar, Dhanya K.Casolani, NicolaNanjappa, DeepakCasperson, ShanonNanno, MasanobuCassarino, MaricaNarasimhulu, Chandrakala AlugantiCassidy, SophieNarayanan, AnandCastellazzi, Anna MariaNaruishi, KojiCastro-Aceituno, VeronicaNascimento, Gustavo G.Catalano, AntoninoNasef, Noha AhmedCauli, OmarNatarajan, Sathish KumarCavazza, NicolettaNath, ArijitCeli, PietroNaughton, ViolettaCena, HellasNaumovski, NenadCenik, BasarNavarrete-Muñoz, Eva MCeranowicz, PiotrNavarro, Carlos Javier GonzálezCermeno, MariaNavarro, VirginiaCesareo, RobertoNavarro-Alarcon, MiguelChae, HeeyoungNavas-Carretero, SantiagoChai, Sheau ChingNaylor, Patti-JeanChakraborty, RajaNeale, RachelChalla, DilipNeeland, MelanieChambers IV, EdgarNegro, MassimoChamcheu, Jean ChristopherNeilson, AndrewChamulitrat, WaleeNelson, GreggChan, CatherineNemoto, KiyomitsuChan, Julie Y.H.Nemzer, Louis RChandra-Hioe, MariaNenna, RaffaellaChandran, ArunaNestel, PaulChandrashekhar, KshipraNetting, MerrynChang, Jer-MingNeves, Adriana CunhaChang, Jung-SuNewby, RuthChao, Pi-YuNewman, John C.Chaplin, AliceNewman, LisaChappell, A.J.Newsom, SeanChappell, AndrewNexø, EbbaChapple, SarahNey, Denise M.Chassard, ChristopheNeymotin, FlorenceChatterjee, JhinukNg, DominicChee, MelissaNg, Qin XiangChen, BoNguyen, PhuongChen, Chung-YiNi Lochlainn, MaryChen, Chun-JungNichols, Buford L.Chen, Guan-WenNicklas, ThereseChen, GuibingNiedzwiecki, MeganChen, GuoxunNielsen, ForrestChen, Kuan-FuNielsen, Forrest HaroldChen, Kuo-HuNieman, David C.Chen, Ling-WeiNikolakakis, IoannisChen, Miaw-LingNinomiya, KiyofumiChen, ShuaiNishi, NobuoChen, WeiqinNishio, Shin-IchiChen, Wei-YuNissensohn, MarielaChen, WenchunNivedita, NiveditaChen, YanaNobles, JamesChen, Yung-ChihNoce, AnnalisaChen, Yung-HsiangNoguchi, TakuyaChen, Yun-WenNolan, JohnChen, ZheNolan, Vikki G.Chen, ZhihangNolden, AlissaChénais, BenoitNomikos, TzortzisCheng, I-ShiungNonaka, KojiCheng, Juei-tangNonogaki, KatsunoriCheng, Xian-wuNoratto, Giuliana D.Chengwen, SunNorman, ÅsaCherbuy, ClaireNorman, TrevorCherkaoui Malki, MustaphaNorsa, LorenzoCherniack, Evan PaulNorval, MaryChertok, Ilana R. AzulayNowak, AdrianaCheskin, LawrenceNowak, Elizabeth CChetwynd, AndrewNowak, Felicia V.Chevalier, StephanieNowicka, GrażynaChiang, Meng-TsanNucci, AnitaChiarioni, GiuseppeNucci, DanieleChiavaroli, LauraNugent, AnneChiba, HitoshiNuytemans, KarenChiefari, EusebioNuzziello, NicolettaChien, Yi-wenNwanodi, OromaChieppa, MarcelloNybacka, SannaChilibeck, PhilO’Halloran, Siobhan A.Chirumbolo, SalvatoreO’neill, Catherine A.Chisholm, AlexandraOaks, Brietta M.Chitchumroonchokchai, ChureepornOba, ShigeyoshiChitraju, ChandramohanObana, AkiraChmurzynska, AgataObanda, DianaChmurzyńska, AgataOberhelman, SaraCho, ClaraObermüller, NicholasCho, ClareObrador, ElenaChobot, VladimirO’Brien, ClaireChoi, Hyung-KyoonO’Brien, Katie M.Choi, LillianOcampo, DiegoChoi, Myung-SookOchoa, TheresaChoo, JinaO’Connor, EibhlísChoo, Pei LingO’Connor, LaurenChoudhary, Kumari SonalOda, EijiChoudhary, SanjeevOgbourne, StevenChoudhury, MalayOh, Yoon SinChouinard-Watkins, RaphaëlOhashi, NaroChoung, Se-YoungOhlsson, BodilChristensen, KristaOhly, HeatherChristians, JulianOhnishi, MasatoshiChristians, UweOho, TakahikoChristiansen, HannaÖhrvik, VeronicaChristides, TatianaOhta, YoshijiChristmann, ViolaOjima, KoichiChristodoulou, Maria-IoannaO’Keefe, Stephen J.D.Christoph, MaryOken, EmilyChristophersen, ClausOkleštková, JanaChruszcz, MaksymilianOkuda, MasayukiChu, Ching-LiangOkuda, NagakoChu, FilmerOkumura, TadayoshiChu, ShidongOlek, RobertChun, Ock KOlendzki, BarbaraChun, ReneOlišarová, VěraChurchward-Venne, TylerOlivecrona, GunillaChurchward-Venne, Tyler A.Oliveira, Paula Alexandra Martins DeChuyen, Hoang V.Oliviero, TeresaCiaccio, MarcelloOlsson, ViktoriaCiappolino, ValentinaOmagari, KatsuhisaCibulskis, CatherineOmar, Syed HarisCiccocioppo, RacheleOmaye, StanleyCiccone, Marco MatteoOmboni, StefanoCicero, ArrigoOmidian, KosarCicero, Arrigo Francesco GiuseppeOmotayo, MoshoodCilla, AntonioO’Neal, EricCione, ErikaOnufrak, StephenCiregia, FedericaOnyenwoke, Rob UClanchy, FelixOoi, KeithClark, Cain C TOpattova, AlenaClarke, MichaelOpie, RachelleClarke, NeilOrdonez Mena, JoséClarys, PeterOrellana, LilianaCleary, RebeccaOrenes-Piñero, EstebanClemens, RogerOsada-Oka, MayukoClemmensen, ChristofferOsorio, JohanClifford, MichaelOstendorf, DanielleClifton, PeterÖstenson, Claes-GöranClimie, RachelO’Sullivan, MariaCoan, P. M.O’Tierney-Ginn, Perrie FCochran, BlakeOtis, Jeffrey S.Coe, Christopher L.Oughton, MatthewCoffey, Patricia S.Oukka, MohamedCohen, RonaldOverwyk, Katherine A.Cohen, Tamara R.Ozawa, KoichiroColaizzo-Anas, TinaOzawa, MioCollado, Pilar SánchezPace, FabioCollado-Yurrita, LuisPace, NelsonColleluori, GeorgiaPacella, ElenaColleran, Heather L.Packiam-Dayalan, AntonyCollier, JasonPadhi, EmilyCollins, JamesPaduch, RomanCollins, Kelsey H.Pagonopoulou, OlgaColonna, GiovanniPal Choudhuri, ShreoshiColson, NataliePalacios, CristinaColumbus, Daniel APalaniappan, BalasubramanianCombet, EmiliePalanisamy, ArulselvanComelli, ElenaPaleari, MicheleComerford, KBPalei, AnaComerford, Kevin B.Paley, Elena L.Comino, IsabelPali-Schöll, IsabellaComstock, SarahPaller, ChanningComulada, Warren ScottPalomba, LetiziaConnaughton, Victoria P.Palomino, Olga MaríaConrad, ZachPalumbo, RoccoConti, PioPalumbo, Vincenzo DavideCooke, MatthewPan, MeixiaCooke, Matthew B.Pan, PanCook-Mills, JoanPanchal, Sunil KCooper, SimonPanek-Shirley, Leah MCooperstone, JessicaPanza, FrancescoCorazzari, MarcoPaoli, AntonioCorbi, GraziamariaPapaccio, GianpaoloCordaro, MarikaPapadakis, SophiaCordero, PaulPapadimitriou, KonstantinosCordero-Herrera, IsabelPapadopoulou, AlexandraCornish, StephenPapet, IsabelleCornu, CatherinePapetti, AdeleCorpe, ChristopherPapi, AlessioCorrêa, Camila RenataParajuli, RajendraCorrea, MariaParhofer, Klaus G.Corsetti, GiovanniPark, Clara YongjooCosta, Ricardo J. SPark, EunyoungCostabile, AdelePark, Jun-BeomCostantini, SusanPark, MarkCostello, Rebecca BortzPark, Sang WonCoughlan, MelindaPark, SimonCoulson, SamanthaPark, Woo JungCox Sullivan, SheilaParker, HelenCox, David N.Parlesak, AlexandrCoxson, PamelaParnell, JillCrawford, LindseyParodi, EmiliaCrawford, SeanParr, EvelynCrenn, PascalParrettini, SaraCresci, Gail AParry, SionCrespo, IreneParsanathan, RajeshCroese, JohnPasanisi, PatriziaCroft, KevinPascual-Teresa, SoniaCroniger, ColleenPasiuakos, StefanCrosby, KarenPasqualetti, GiuseppeCross, Carroll E.Pasqualone, AntonellaCrowe, FrancescaPassamonti, SabinaCubero, JavierPaßlack, NadineCuberos, Ramón ChacónPastori, DanieleCui, ChuanlongPatankar, Manish S.Cullere, MarcoPatarrão, RitaCusanelli, EmilioPateiro, MirianCzaja-Bulsa, GrażynaPatel, DevenCzikora, IstvanPatel, HardikkumarCzogalla, KatrinPatel, Harnish P.Czyżowska, AgataPatel, PriyankaDa Paz, Vanessa Ribeiro FigliuoloPatel, Yashomati M.Da Silva, Robin P.Pathakoti, KavithaDachs, GabiPatridge, StephanieDahdah, LamiaPatrignani, FrancescaDahl, CeciliePatterson, KiraDahl, LisbethPatti, Mary-ElizabethDai, ZhaoliPaul, DavidDaimiel, LidiaPavlov, TengisDal Ben, MatteoPazzagli, LuigiaDalbeni, A.Pearce, ElizabethD’Alessio, Patrizia A.Pearson, NatalieDall’Acqua, StefanoPeart, DanielDall’Asta, ValeriaPeckham-Gregory, Erin C.Dalmasso, MarionPedersen, Kim B.Dalton, BethanPedroni, MarcoDalton, CarolinePeinado, Juan R.Dan Ramdath, D.Pekkinen, MinnaDando, RobinPeña, Amado SalvadorDanezis, GeorgiosPeng, LifengDao, Maria CarlotaPeng, MeiDarcy, JustinPeng, WenboDarling, Andrea LPenniston, Kristina L.Darmaun, DominiquePenugonda, KavithaDarmon, NicolePepe, JessicaDarnton-Hill, IanPepeu, GiancarloDas, AditiPereira, Carlos DiasDas, UndurtiPereira, Cláudia Maria F.Dash, RanjeetPereira, DoraDatta, PoulamiPereira-da-Silva, LuisDaugelavicius, RimantasPereira-da-Silva, LuísD’Auria, EnzaPérez-Burillo, SergioDaussin, Frédéric NicolasPérez-Cano, Francisco J.Dave, Kunjan R.Perez-Cornago, AuroraDavey, AdamPerez-Cueto, ArmandoDavila, Anne-MariePérez-Gálvez, AntonioDavinelli, SergioPerez-Jimenez, JaraDavison, KarenPérez-López, AlbertoDavy, BrendaPérez-Matute, PatriciaDe Baaij, JeroenPérez-Rodrigo, CarmenDe Berardis, DomenicoPerez-Stable, CarlosDe Blasio, Miles J.Periago, María JesúsDe Deurwaerdère, PhilippePero, RaffaelaDe Geest, BartPerrier, JosetteDe Gruijl, FrankPerrine, CriaDe La Rosa, Laura A.Perry, TracyDe Lange, PieterPeters, John C.De Las Heras, JavierPeters, Rachel L.De Magistris, LauraPetersen, KristinaDe Paepe, BoelPetersen, Kristina S.De Paula Venancio, ViniciusPeterson, Catherine ADe Sensi, FrancescoPeterson, JuliaDe Seymour, JamiePeterson, LeighDe Vadder, FilipePetriello, Michael CDe Vita, ValerioPetro, Thomas M.De Vito, ElisabettaPfohl-Leszkowicz, AnnieDe Vries, JanPhang, MelindaDe Vries, JeannePhilips, NeenaDe Wet, HeidiPhinney, Karen W.Dean, ElizabethPi, XinchunDe-Bandt, Jean-PascalPiancatelli, DanielaDeBiasse, Michele A.Piccoli, GiorginaDeClercq, VanessaPiccolo, BrianDeer, RachelPiche, Marie-EveDekker, MarloesPicklo, Matthew J.Del Giudice, RitaPiemontese, LucaDel Mistro, AnnarosaPiernas-Sanchez, CarmenDel Rio, DanielePietrobelli, AngeloDelarue, JacquesPignitter, MarcDella Torre, SaraPilkington, Suzanne M.Dellavalle, Diane M.Pinent, MontserratDelmastro-Greenwood, MeghanPinheiro, MarinaDelvin, Edgard E.Pinto, AlessandroDen Hartigh, Laura J.Pinto-Sanchez, Maria InesDenham, Bryan E.Pirisi, LuciaDenisow, BozenaPirola, LucianoDepalma, Ralph G.Piu, SahaDerave, WimPiva, TerrenceDerman, OrhanPlastina, PierluigiDesai, AbhishekPlaydon, MaryDesautels, Danielle N.Plaza-Díaz, JulioDeschasaux, MelaniePlaza-Zabala, AinhoaDeshpande, GirishPlochocki, JeffreyDeth, Richard C.Plogsted, SteveDetzel, PatrickPlummer, VirginiaDeutsch, JonathanPochi, SubbarayanDev, DiptiPoder, Thomas G.Devereux, StephenPognonec, PhilippeDevine, AmandaPogorzelska-Nowicka, EwelinaDevries-Aboud, MichaelaPohl, SebastianDeWeese, RobinPoindexter, BrendaDey, MoulPolanska, KingaDhakal, DipeshPolidori, M. CristinaDhillon, JaapnaPollock, Richard L.Di Biase, StefanoPomeroy, JeremyDi Carlo, MartaPons, AntoniDi Daniele, NicolaPoole, David C.Di Donato, MarziaPopeijus, Herman E.Di Felice, GabriellaPopłoński, JarosławDi Giannantonio, MassimoPopovich, DavidDi Gioia, DianaPorres, JesusDi Iorio, Biagio RaffaelePorter, Judi A.Di Lorenzo, CherubinoPotter, JuliaDi Mauro, Maria DomenicaPotter, MichaelDi Nunzio, MattiaPoudel, BarunDi Primio, RobertoPowell, LisaDi Tola, MarcoPowers, Jacquelyn MDiamanti, AntonellaPowles, JohnDíaz, LorenzaPrabhu, SandeepDibaba, DanielPrakasam, SivaramanDickerson, RolandPravst, IgorDickinson, KaciePrentice, HowardDieleman, LevinusPrice, Ramona SalcedoDieterich, WalburgaPrietl, BarbaraDietrich, Johannes W.Prieto, IsabelDijkstra, GerardPrimeaux, Stefany D.Dilger, RyanPrince, BennieDillon, Edgar LicharProbst, YasmineDimas, KonstantinosProcter, Sandra B.Dimova, ElitsaProdam, FlaviaDing, LinProf Bouscher, BarberaDing, YinyuanProietto, JosephDingemans, AlexandraProkesch, AndreasDJ, MillwardProtudjer, JenniferDoddapaneni, RaviPrykhodko, OlenaDoddapattar, PrakashPsurski, MateuszDoerr, CelestePuertas Molero, PilarDoig, Gordon S.Pulido, Olga MDolores Marrodán, MaríaPulker, Claire ElizabethDomínguez, RaulPundir, ShikhaDomínguez, RaúlPursey, KirrillyDonadio, CarloQasem, Wafaa A.Donato, RosarioQian, GuoqingDongiovanni, PaolaQian, QiDonne, BernardQin, JuanD’Onofrio, GraziaQin, LiqiangDonofry, Shannon D.Quadros, Edward V.Doo, MiaeQuaresma, Mário A.G.Dordevic, AimeeQuigley, EamonD’Oria, LuisaQuinn, Mark T.Doriano Bianco, ArmandoQuintero-Florez, AngelicaDorling, JamesR Bruns, DanielleDos Santos Niculau, EdenilsonRaatz, Susan K.Doyle, LornaRaben, Anne B.Dreher, MarkRacette, SusanDreher, Mark L.Rachek, LyudmilaDrevon, Christian ARachek, Lyudmila I.Drobnic, FranchekRadmacher, PaulaDrouin-Chartier, Jean PhilippeRafiei, HosseinDu, CaiganRafiq, ShamailaDu, MinRagusa, AndreaDuarte, T. L.Raheem, BamideleDucatelle, RichardRahimi, FaridDuconseille, AnneRahimi, Robert S.Dudas, JozsefRahman, MilladurDugast, PascaleRai, PrakashDUmeUS, StanleyRai, VikrantDumler, FrancisRaikou, Vaia D.Duncanson, KerithRainger, George EdwardDuntas, LHRaj, Madhwa H.G.Durazzo, AlessandraRajaram, SujathaDurgan, David J.Rajendran, PraveenDurkalec-Michalski, KrzysztofRalle, MartinaDurward, CarrieRalli, MassimoDusso, AdrianaRallis, MicaelDuwaerts, CarolineRalston, Nicholas V. C.Dyck, David J.Ralston, PennyEarnest, ConradRamachandiran, IyappanEbrahim, WeaamRamadas, AmuthaEck, Kaitlyn M.Ramadori, GiulianoEda, ShigetoshiRamakrishnan, SadeeshEdurne, Simón MagroRaman, JayEdwards, Adrianne N.Ramdas, Wishal D.Edwards, CathrinaRamich (Pivovarova), OlgaEdwards, Joshua R.Ramírez-Vélez, RobinsonEdwards, SarahRamkumar, VickramEfird, JimmyRamos, Joyce S.Egger, GarryRamos, SoniaEiby, YvonneRangan, AnnaEilertsen, Karl-ErikRangan, GopiEisenga, Michele F.Ranzato, EliaEkpeni, LeonardRao, VidhyaEl Ghoch, MarwanRasmussen, HeatherEl Khoury, DaliaRathi, NehaEl Midaoui, AdilRathinasabapathy, AnandharajanEl Muayed, MalekRauch, BernhardElam, MarcusRaval, ManmeetElango, RajavelRaverdeau, MathildeEldeeb, MohamedRawling, TristanElder, GrahameRawson, AshishEleftheriadis, TheodorosRaz, OlgaEl-Esawi, MohamedRazzaque, MohammedElisabet, ForsumReale, MarcellaEllervik, ChristinaReboul, PascalElli, LucaRedan, BenjaminEllinger, SabineReddy, Prem VeerElliott, Hannah RReddy, SakamuriElmi, AlbertoReeve, VivienneElshiewy, OssamaRehm, Colin DEmerson, Dawn M.Reicks, MarlaEmilsson, LouiseReidlinger, DianneEmond, Jennifer A.Reidy, PaulEndesfelder, StefanieReig García-Galbis, ManuelEnjapoori, Ashwantha KumarReiger, MatthiasErdman, JohnReiner, MartinEri, RajReiter, E. MirandaEroğlu, AbdulkerimRemacha, Ángel F.Ershow, AbbyRemaley, Alan T.Ershow, Abby G.Remer, ThomasEscolà-Gil, Joan CarlesRemesar, XavierEslam, MohammedRenner, WilfriedEsmaili, SaeedRepa, AndreasEspada, JesúsResch, UlrikeEspino, Daniel M.Rescigno, AntonioEsposito, CiroReunala, TimoEsquerra-Zwiers, AnitaRevuelta Iniesta, RaquelEstanis, NavarroReynoird, NicolasEsteve-Romero, JosepReynolds, Andrew N.Esteves, EduardoReynoso, ElenaEstruch, RamonRezzani, RitaEto, BrunoRho, JongEvans, E WhitneyRhoads, J. MarcEvans, Franklin D.Rhodes, JonathanEvans, GethinRibaldone, Davide GiuseppeEvans, Joseph L.Ribas, GloriaEvans, MaryRibeiro, RosileneEverts, HelenRibot, JoanEyles, Darryl W.Ricci, AriannaEze, Ikenna C.Ricci, Elena ClaireEzura, YoichiRiccio, PaoloFabiani, RobertoRice, Madeline MurguiaFabre, ClaudineRichard, Erin L.Fadok, ValerieRichter, JörgFahey, GeorgeRienecke, ReneeFahey, JedRigo, JacquesFairweather, StephenRijkers, GerFajardo, RobertoRingel-Kulka, TamarFajardo, Val AndrewRink, LotharFalasca, MarcoRios-Covian, DavidFaldyna, MartinRitze, YvonneFalkenstein, MichaelRivera, EleanorFallaize, RosalindRivero-Pérez, M. DoloresFallows, StephenRizzello, CarloFan, QiongRizzo, GianlucaFan, XiuchengRizzo, GiuseppeFarebrother, JessicaRobberecht, HarryFarhat, GraceRobertson, KirstenFarzaneh, VahidRobinson, Daniel T.Fasinu, Pius S.Robinson, EdwardFaure, SébastienRobinson, EricFava, CristianoRobledo, Virginia RodríguezFava, Cristiano C.Robson, Shannon M.Favero, GaiaRockwell, MichelleFayet-Moore, FlaviaRodrigues, Celia F.Feas, XesusRodríguez Villanueva, JavierFedele, FrancescoRodriguez, Carly AliciaFedosov, SergeyRodriguez, Francisca SFeinman, RichardRodriguez-Herrera, AlfonsoFelder, Robin A.Rodriguez-Mateos, AnaFeng, Zhi ChaoRoe, LianeFeresin, Rafaela G.Roepke, TroyFerguson, BradleyRogers, LisaFernández A., LeónidesRogers, Lynette K.Fernández, IgnacioRogerson, DavidFernandez, Maria LuzRoh, ChanghyunFernández-Mateos, PilarRohner, FabianFernández-Sánchez, M.L.Rokavec, MatjazFernando, BinoshaRolan, PaulFerrante, ClaudioRolandsdotter, HelenaFerraro, Pietro ManuelRomani, AndreaFerrie, SuzieRomero Martínez, ÁngelFetissov, Sergueï O.Ronkainen, JustiinaFezeu, LeopoldRoodenburg, AnnetField, Martha S.Roodenrys, StevenFielding, BarbaraRoot, MartinFields, DavidRosaly, Correa-de-AraujoFilannino, PasqualeRoselli, MariannaFiocco, DanielaRosendahl-Riise, HanneFioravanti, AntonellaRoshanbinfar, KavehFischer, FlorianRosi, AliceFiset, SylvainRosner, BernardFisher, GordonRosset, RobinFisher, JeffreyRossi, Andrea P.Fitsanakis, VanessaRossi, FilippoFlack, JohnRossi, PaolaFlack, Kyle D.Rostami, KamranFlax, ValerieRoubinian, NaregFletcher, EllyRouch, AlFlexeder, ClaudiaRouhiainen, AriFlinn, JaneRousseau, GuyFlynn, Mary M.Roy Choudhury, GouravFlythe, MichaelRoy, BimolFocsan, Alexandrina L.Roy, Brian D.Fogacci, FedericaRoy, DenisFölster-Holst, ReginaRoychowdhury, SanjoyFontés, MichelRubio, LauraFoong, Rachel E.Rubio-Arraez, SusanaForbes, ScottRudkowska, IwonaForbes, TamaraRudolph, Michael C.Forhead, Alison J.Rufián-Henares, José ÁngelForman, MicheleRuiz, MarioForsyth, AdrienneRuiz-Barquín, RobertoFortenberry, MeganRuiz-Moreno, EmmaFoskett, AndrewRuiz-Robledillo, NicolásFoster, Charles B.Ruoppolo, MargheritaFoster, EmmaRusciano, DarioFouladkhah, AliyarRuscica, MassimilianoFourtounas, CostasRush, Elaine CFoutch, BrianRush, Elaine C.Fox, SimonRuss, JohnFragkos, KonstantinosRussell, Fraser D.Frame, Leigh A.Russell, MarkFrancis, HeatherRyabov, IgorFrank, JanRyan, Elizabeth P.Frank, SašaRybak, LeonardFranklin, PeterS. Lopez, DavidFranko, AndrasSaavedra, Jose M.Franzè, AnnamariaSabia, MichaelFraser, DavidSaccà, Sergio C.Frazzi, RaffaeleSadanaga, TsuneakiFrediani, JenniferSaeedi, PouyaFreedman, MarjorieSafonte, FabiolaFreisling, HeinzSagayama, HiroyukiFresco, PaulaSagnou, MarinaFreund, NadjaSaiardi, AdolfoFriel, JamesSaikumar, PothanaFriesen, CarolSainsbury, KirbyFritsche, KevinSaisho, YoshifumiFrosini, MariaSaito, YoshiroFrossard, Jean-PierreSaito, YukihiroFruehbeck, GemaSajdakowska, MartaFu, QiangSakamuri, Siva Sankara Vara PrasadFu, XueyanSakuma, KunihiroFu, YuSakurai, ToshihiroFueyo-Díaz, RicardoSalaheen, SerajusFujimori, KoSalazar, GloriaFujita, SatoshiSalazar-Villanea, SergioFujita, TetsujiSaldanha, CarlotaFukuda, MitsuruSaldanha, SabitaFukui, AtsushiSaleem, AyeshaFulford, JonathanSalinero, Juan JoséFulgoni, Victor L.Salles, ChristianFunaba, MasayukiSallis, AmandaFung, Ellen B.Salonen, Bradley R.Furman, LydiaSaluk-Bijak, JoannaFürnsinn, ClemensSalvatore, MirellaFusaro, MariaSalvestrini, CamillaG Gamage, DilaniSalvia, RosannaGaddam, Ravinder ReddySalvo, AndreaGaesser, GlennSamala, Niharika R.Gai, FrancescoŠamec, DunjaGaikwad, RaviSampathi, ShilpaGaitán, Adriana V.Samsoondar, JoshuaGalanis, AlexisSamuelsson, Anne M.Galbete, CeciliaSanada, KiyoshiGaleazzi, Gian MariaSánchez, Manuel CastroGali, HimabinduSánchez-Fidalgo, SusanaGalloway, StuartSánchez-Muniz, Francisco José J.Galvez, JulioSanchez-Oliver, Antonio JGanapathy, VadivelSandberg, Ann-SofieGander, JenniferSanders, ThomasGandy, JoanSangelaji, BahramGangloff, AnneSankavaram, KavithaGangola, Manu PratapSanmartin, ChiaraGanguli, Kriston A.Santamaria, RitaGanji, VijaySantanasto, Adam J.Gannon, BryanSantangelo, CarmelaGänzle, Michael G.Santos López, Jorge ArturoGao, JieSantos, DeboraGao, MingmingSantos, Rafael BravoGarcía De Frutos, PabloSantos-Juanes, JorgeGarcia Layana, AlfredoSantovito, ElisaGarcía-Ayuso, DiegoSantulli, GaetanoGarcia-Bailo, BibianaSanz-Paris, AlejandroGarcía-Conesa, María-TeresaSaotome, MasaoGarcía-Estañ, JoaquínSapone, AnnaGarcia-Pardo, JavierSaraiva, NunoGarcía-Ródenas, ClaraSardão, VilmaGarcía-Sánchez, AsunciónSarkar, AmritaGard, PaulSarma, SauravGardener, SamanthaSarris, JeromeGardner, Andrew S.Sasaki, TsutomuGardner, Kevin H.Sato, KenjiGardner, ReneeSato, ShinGarin, NoéSatsu, HideoGarnacho-Castaño, Manuel VicenteSauma, DanielaGarnweidner-Holme, LisaSautto, Giuseppe A.Garrett, Greg S.Savastano, SilviaGarrote, Jose AntonioSavi, MoniaGartner, Lawrence MSavino, FrancescoGarza, KimberlySavoie Roskos, MatejaGasana, JanvierSaw, Constance L.L.Gavrieli, AnnaSawada, NorieGay, MelvinSawicki, RafalGay-Quéheillard, JeromeSayer, R. DrewGe, DongxiaSayón-Orea, CarmenGe, XiaodongScarano, AureliaGeisler, CorinnaScazzina, FrancescaGenoni, AngelaSchaafsma, A.Georgiev, VasilSchaardenburg, D. VanGeraghty, PatrickSchachter, MichaelGerber, Leonard E.Schaffer, StephenGerber, MarietteSchenk, JeannetteGermano, PatriziaScherf, KatharinaGeurts, JeroenSchicho, RudolfGevers, Dorus W.M.Schiraldi, ChiaraGhag, GauravSchirmer, BastianGharaibeh, BurhanSchlaepfer, Isabel RGhatak, ArindamSchlaff, Rebecca AnnGholami, MaryamSchleinitz, DoritGhosh, SumitSchlieper, GeorgGiacomelli, ChiaraSchloss, Janet M.Giacomello, EmilianaSchmidt, RicardaGiampieri, FrancescaSchmidt-Arras, DirkGianni, Maria LorellaSchnackenberg, LauraGiannopoulou, IfigeneiaSchoeler, Natasha E.Giansanti, FrancescoSchoeller, DaleGiapros, VasileiosSchoenaker, Danielle AJMGibbons, JeffSchöpfer, JuttaGibbs, Bernhard F.Schott, UlfGibbs, HeatherSchrager, MatthewGibson, Alice ASchram, BenGibson, AnnSchubert, MattGibson, RachelSchubert, Matthew M.Gibson, SigridSchürmann, AnnetteGiezenaar, CarolineSchwab, UrsulaGifford, Janelle A.Schwarz, PeterGiles, CoreySchwetz, VerenaGiles, Grace E.Scicchitano, PietroGill, TiffanyScoditti, EgeriaGillevet, PatrickScoffield, JessicaGillis, DorisScorziello, AntonellaGiltay, ErikScott, Fraser W.Giombini, LuciaScott, HaleyGiovambattista, AndresSeale, LuciaGiovannoli, CristinaSebastian, AnthonyGiraldo, AlejandroSeca, Ana Maria Loureiro DaGiromini, CarlottaSeconda, LouiseGiron, Maria CeciliaSedlacek, AbigailGist, NicholasSeekatz, Anna MariaGiudetti, AnnaSeeman, Mary V.Giuseppe, Romina DiSeetharaman, ShyamGkimpas, GeorgiosSegovia-Siapco, GinaGłąbska, DominikaSegura-Garcia, CristinaGlastras, SarahSeimon, RadhikaGlauert, Howard P.Sekikawa, AkiraGlinz, DominikSellayah, DyanGlynn, Erin L.Sempos, ChrisGodos, JustynaSemrau, KatherineGoel, PeeyushSenesi, PamelaGoer, MaureenSengupta, ArjunGoetz, MargaretheSeo, Dong HoGogou, MariaSeo, Young AhGogulamudi, Venkateswara ReddySerafini, GianlucaGois, Pedro FrancaSerpanos, YulaGolczak, MarcinSerra, Ana TeresaGolmohamadi, AmirSerra, DolorsGomez De Agüero, MercedesSerra, FranciscaGómez Llorente, CarolinaSerra, GessicaGómez-Gallego, CarlosSerra, MonicaGomez-Pinilla, Pedro J.Serrano, Jose C. E.Gomez-Salgado, JuanSestak, KarolGomollon, FernandoSestili, PieroGondalia, Shakuntla V.Seth, DevanshiGong, XiaomingSeth, RataneshGonzález Valero, GabrielSeuter, SabineGonzález, Concepción C.Seyfried, Thomas N.Gonzalez, JavierSha, ZheGonzález, SoniaShah, AjitGonzalez-Barcala, Francisco JavierShah, Rachana D.González-González, RogelioShahar, DanitGonzález-Minero, Francisco JoséShaish, AvivGonzález-Navarro, HerminiaShakibaei, MedhiGonzález-Stuart, ArmandoShankar, EswarGonzalez-Vicente, AgustinShankar, PadminiGoodman, Richard E.Shao, AndrewGoodman, SamanthaShapiro, Allison L. B.Gordon, Eliza L.Shapiro, Allison L.B.Gorham, EdwardSharma, AtulGorman, ShelleySharma, BheshGorrell, SashaSharma, Bhesh RajGorska-Ponikowska, MagdalenaSharma, IshaGórska-Warsewicz, HannaSharma, NaveenGosselet, FabienSharma, RohitGoswami, NanduSharma, UmakantGoto, YoshiyukiSharples, AdamGoua, MarieShaw, OdetteGoulet, EricShea, KylaGow, Megan L.Shearer, Gregory C.Gowda, MadanShearrer, Grace EGower, BarbaraSheikh, AlaullahGraça, JoãoShen, Szu-ChuanGrace, Mary HalimShen, ZhenyuGrace-Farfaglia, PatriciaShertzer, Howard G.Graef, JenniferSheth, SandeepGrafenauer, SaraShewale, SwapnilGramlich, LeahShiao, S. Pamela K.Granados-Principal, SergioShidoji, YoshihiroGranfors, MichaelaShigeru, YatohGranic, AntonetaShimizu, YukihiroGrant, GeorgeShimosawa, TatsuoGrases, FelixShin, AndrewGrasselli, ElenaShindou, HideoGrattagliano, IgnazioShivakoti, RupakGray, AndrewShivappa, NitinGrech, AmandaShoben, Abigail B.Green, PeterShoenfeld, YehudaGreen, TimShokal, UpasanaGreen, Timothy JohnShriver, LenkaGreene, Michael W.Shukla, Alpana P.Greening, ChrisShukla, ShrutiGreening, StevenShypailo, Roman J.Greer, Frank RSiatka, TomášGregori, DarioSibilla, SaraGreyling, ArnoSicinska, EwaGrgic, JozoSidhu, RohiniGrider, ArthurSieber, FritzGridneva, ZoyaSiebers, RobGrieger, Jessica ASiegel, Robert M.Griffiths, Helen R.Sierpina, VictorGrilli, EsterSies, HelmutGrimm, Marcus OWSilswal, NeerupmaGrisk, OlafSilveira, Patrícia P.Groh, Isabel Anna MariaSim, AaronGropper, SareenSimes, DinaGross, Susan MichelleSimões-Wüst, Ana PaulaGrossini, ElenaSimon, Marie-ChristineGrosso, GiuseppeSimoni, JanGrothusen, ChristinaSimons, Courtney WayneGruengreiff, KurtSimper, TrevorGrzeskowiak, Luke ESimpson, Melanie RaeGu, FengSinclair, AndrewGu, YianSingh, BanoGualdoni, GuidoSingh, ChandraGuan, WenjianSingh, NagendraGuandalini, StefanoSingh, SudhaGuasch-Ferre, MartaSingh, VishalGudbrandsen, Oddrun A.Singla, BhupeshGudey, Shyam KumarSiniscalco, DarioGuéant, Jean LouisSirover, William DGueimonde, MiquelSirtori, CesareGuevara, Pilar EgüezSiska, Peter J.Guido, Luis F.Sita, GiuliaGuinane, CaitrionaSivakumar, SushamaGuldris, Secundino CigarranSivaprakasam, SathishGunasekar, PalanikumarSjögren, KlaraGunst, JanSkeaff, SheilaGunton, JennySkenderi, Katerina PGuo, WeiminSkopouli, Fotini NikolaosGuppy, FergusSkypala, IsabelGupta, KajalSlater, JoyceGustafsson, Ulf O.Slyvka, YuriyGutierrez, J. ClaudioSmetana, SergiyGutiérrez-Juárez, RogerSmeuninx, BenoitGuzek, DominikaSmith, ClaireGuzzardi, Maria AngelaSmith, GordonGyamfi, Maxwell A.Smith, JohnEricGyawali, PrajwalSmith, KennethH Shirvani, ArashSmith, KylieH. Gilhooly, CherylSmith, LeeHaase, HajoSmith, MoiraHaboubi, NadimSmith, Robert E.Hackett, DanielSmith, TarynHackman, Robert M.Smrke, SamoHaddow, James EdwardSnow, DeniseHair, AmySoares, Mario J.Hair, Amy B.Sobczyńska-Malefora, AgataHajduch, EricSöderström, LisaHaldar, SumantoSoe, KentHallmann, EwelinaSoenen, StijnHalloran, Bernard P.Sogari, GiovanniHam, DanielSoglia, FrancescaHamaguchi, MasahideSokal-Gutierrez, KarenHambidge, K. MichaelSokół-Łętowska, AnnaHametner, BernhardSolanki, NaimeshHamlin, RobertSommer, AlfredHampel, DanielaSone, HidekoHamulka, JadwigaSong, JiajiaHan, JaehongSong, MingHan, Seung JinSong, Sang-WookHan, Sung NimSong, SuHan, ZhiyongSong, YoonjuHanson, Corrine KSopade, Peter A.Hanson, Karla L.Sosna, JustynaHao, JiaqingSotomayor, Camilo G.Hao, LeiSotos-Prieto, MercedesHarada, NaokiSoulika, AthenaHaramoto, EijiiSoumyanath, AmalaHarbron, JanettaSpadafranca, AngelaHarcourt, BrookeSpagnuolo, RoccoHardman, Roy J.Spanova, AlenaHare, DominicSpence, DavidHaring, BernhardSpiegler, JulianeHarnack, LisaSpiegler, VerenaHarnett, JoannaSpinelli, SaraHaron, MonaSpitznagel, Mary BethHarris, CarlaSpoto, BelindaHarris, MargaretSrinivasan, VijayHarris, Philip J.Srivastava, SarikaHarris, Randall ESrivastava, SaurabhHarrison, FionaSt Hilaire, MelissaHarrison, Jonathan S.St Hill, CatherineHarroun, ThadStacewicz-Sapuntzakis, MariaHarthoorn, Lucien F.Stadlbauer, VanessaHarting, JannekeStamm, Rosemary A.Harton, AnnaStandl, MarieHaskell-Ramsay, CrystalStanhope, JessicaHassan, FauziyaStanley, JoannaHassan-Smith, ZakiStanley, Jone A.Haszard, JillStannard, StephenHatsu, IreneStarowicz, MałgorzataHaupt-Jorgensen, MartinStasiewicz, Matthew J.Havel, PeterStauber, Rudolf E.Hawinkels, LukasStaunstrup, Nicklas HeineHawkes, Cheryl A.Stec, David E.Hawkins, KeelySteenson, SimonHay, Alastair W MStefan, NorbertHayes, AlanStefanidou, Maria E.Hayes, JohnStefler, DenesHayes, K C.Stegelmeier, BryanHaytowitz, David BStelmach-Mardas, MartaHeberden, ChristineStephens, Jacqueline M.Hebert, JamesSterken, MarkHebert, James R.Stevenson, EmmaHebestreit, AntjeStewart, Maria L.Hedrick, ValisaStirton, RuthHefferon, Kathleen LauraStiuso, PaolaHeil, Sandra GStobdan, TseringHeinen, MirjamStokes, Caroline S.Hekmatdoost, AzitaStone, Trevor WilliamHelms, EricStoppe, ChristianHelmy, Yosra A.Storcksdieck Genannt Bonsmann, StefanHemilä, HarriStornaiuolo, MarianoHendren, RobertStote, KimHendrich, SuzanneStrand, ElinHenjum, SigrunStrandvik, BirgittaHennigar, StephenStratakis, NikosHenriksson, PontusStrehle, Eugen-MatthiasHernández, AdrianaStremke, Elizabeth R.Hernandez, MariaStrien, Tatjana VanHernandez-Alonso, PabloStringer, AndreaHerndon, DavidStubbs, James R.Herrick, KirstenStull, April J.Hervás, GotzoneSu, Zheng-YuanHetherington, MarionSubar, AmyHetland, GeirSubhan, Fatheema BegumHettinga, KasperSubramanian Vignesh, KavithaHewitt, StephenSugimoto, TaikiHewlings, Susan J.Sugita-Konishi, YoshikoHeydemann, AhlkeSugiyama, KemmyoHeyn, PatriciaSuh, Jun GyoHeyne, HenrikeSui, ZhixianHida, AzumiSukocheva, OlgaHideaki, KashimaSukumar, DeepthaHider, Robert C.Sukumaran, Anuraj TheradiyilHietavala, Enni-MariaSukumaran, Sunil KumarHigashida, KazuhikoSuleria, HafizHigashimura, YasukiSuman, ShubhankarHiggins, MattSun, Hui BHildreth, BlakeSun, JunHill, AileenSun, NaweiHilmers, AngelaSun, QianHimoto, TakashiSun, ShiHind, MatthewSundekilde, Ulrik K.Hirasaka, KatsuyaSung, ValerieHirata, YokoSuriano, FrancescoHirschi, Kendal D.Sutter, CarolynHivert, Marie-FranceSuzuki, KatsuhikoHo, Allen L.Swoap, StevenHobbs, DitteSyed, AkheelHobin, ErinSyed, Deeba NadeemHocher, BertholdSylvetsky, Allison C.Hocquette, Jean-FrançoisSzászi, KatalinHodge, AllisonSzlagatys-Sidorkiewicz, AgnieszkaHoefer, ImoSzumny, Antoni JacekHoeller, UlrichSzychlinska, Marta AnnaHoerr, RobertTaber, ChristopherHoffmanová, IvaTada, HayatoHögger, PetraTadic, VanjaHokama, TomikoTai, Dar-FuHolden, Stephen STain, You-LinHolecek, MilanTajiri, YujiHolefors, AnnaTakahama, UmeoHolick, MichaelTakahashi, NobuyukiHolland, Angelia MaleahTakaki, AkinobuHollis, BruceTakaya, JunjiHolm, LarsTakayama, KenichiHolmstrup, PalleTakayuki, TsukuiHolsinger, DamianTalati, ZenobiaHolt, DarcyTaleb, NadineHolt, RobertaTamura, YukinoriHolzapfel, ChristinaTan, Bee KangHolzmann, KlausTan, BKHomko, Carol J.Tan, IsabellaHong, JaeheeTan, Sih MinHong, QiutingTan, Sze YenHooda, JagmohanTan, XiaoHoogeveen, Ellen K.Tanabe, KatsuyukiHoracek, TanyaTanaka, KeikoHorrigan, Louise A.Tanaka, TakujiHorstmann, AnnetteTanaka, TamotsuHosick, PeterTanasova, MarinaHosokawa, YoshitakaTang, MinghuaHossain, BelayatTanner, S. BoboHouchins, JennyTannous, KathyHouston, Kevin D.Taoka, YousukeHoward Wilsher, StephanieTapia, GermanHowarth, Gordon S.Tappia, Paramjit S.Howe, StephanieTappy, LucHruz, Paul W.Tapsas, DimitriosHsieh, ChangweiTarantino, GiovanniHu, ChaoTarleton, Emily K.Hu, GuochangTarozzi, AndreaHu, JianTarrago, LionelHu, TianTarragon Cros, ErnestoHuab, MarkTasevska, NatashaHuang, Chi-ChangTattikota, Sudhir GopalHuang, Chun-JungTavori, HagaiHuang, Guan-JhongTawia, SusanHuang, XiaohuTay, JeannieHübner, FlorianTayebati, Seyed KhosrowHuedo-Medina, Tania B.Tayie, FrancisHuggins, Catherine E.Taylor, Carla G.Hughes, CatherineTaylor, RachaelHughes, David J.Taylor, Rachael M.Huling, JenniferTe Morenga, LisaHulthén, LenaTeas, JaneHuneau, Jean FrançoisTelang, SuchetaHung, YungTelford, Rohan M.Hunsberger, MonicaTemme, Elisabeth HMHur, Sun-JinTempera, GiannaHuston, Robert K.Temple, NormanHutchinson, JayneTeng, BoHuysentruyt, KoenTeodoro, JoãoHyde, Natalie K.Teotia, PoojaIampietro, MathieuTerpou, AntoniaIatsenko, IgorTeske, JenniferIbrahim, KindaTessari, PaoloIchikawa, HiroshiTessema, MasreshaIchimura, HiroshiTeusch, NicoleIde, KazukiTeves, María EugeniaIdo, YasuoThabrew, HiranIhalainen, JohannaThackray, Alice EmilyIizuka, KatsumiThijs, CarelIkeda, MasayukiThiruvengadam, MuthuIkegaya, NaokiThoene, MelissaIkushiro, Shin-ichiThomas, Diana MariaIlag, Leodevico L.Thomas, TiffanyIlavenil, SoundharrajanThomas, WayneIlmer, MatthiasThomsen, HaukeImai, ToshioThomson, David M.Imamura, FumiakiThornley, SimonImig, JohnThornley-Brown, DenyseImperatore, NicolaThornton, Laura M.Imperatori, ClaudioThyagarajan, BaskaranInamura, KentaroTiao, Mao-MengIngram, DonaldTicha, AlenaInns, StephenTicinesi, AndreaInvitto, SaraTickell, Kirkby D.Ioakeimidis, IoannisTieland, MichaelIp, BlancheTiidus, PeterIrwin, ChrisTimon, Claire MarieIshaq, Suzanne LynnTincati, CamillaIshihara, HisamitsuTintle, NathanIshihara, KengoTober, KathleenIsola, GaetanoToegel, StefanIspoglou, TheocharisToepel, UlrikeItkonen, Suvi T.Toledo-Corral, ClaudiaIto, NaokiTomaro-Duchesneau, CatherineIuliano, RodolfoTomaszewska, EwaIuliano, SandraTong, TammyIves, StephenToniolo, LuanaIwahori, ToshiyukiTonkin, EmmaIwai, AtsushiTonolo, GiancarloIyer, ChitraToorie, AnikaIzawa, Kazuhiro P.Torinsson Naluai, ÅsaIzawa, TakeshiTorquati, LucianaJablonska, EwaTorrella, Joan RamonJabłońska-Trypuć, AgataTorres Fuentes, CristinaJacka, Felice NTorres López, M IsabelJackson, AlanTorres, SusanJacob, NoamTørris, ChristineJacobs, DavidTotosy De Zepetnek, JuliaJacobsen, Elisabeth EgholmToumpakari, ZoiJadavji, Nafisa M.Tourniaire, FranckJafariNasabian, PegahTovoli, FrancescoJagannathan, RamTownsend, JeremyJaggupilli, AppalarajuTraina, GiovannaJahan-Mihan, AlirezaTrakman, Gina L.Jahidul, IslamTrehan, IndiJahn, SteffenTrevena, HelenJahns, LisaTrevisan, AndreaJain, AnshikaTrifilieff, PierreJajtner, AdamTripathi, ManishJakobsen, JetteTripolt, NorbertJala, Venkatakrishna RaoTríska, JanJalili, ThunderTriunfo, StefaniaJames, BryonyTroen, AronJames, PhilipTrojanowicz, BoguszJamieson, Jennifer ATrommelen, JornJanda, ElzbietaTrovato, FrancescaJandacek, Ronald J.Trovato, GuglielmoJang, Byoung KukTrovato, Guglielmo M.Jannasch, FranziskaTroy, Lisa M.Jape, GayatriTrummer, OliviaJaradat, AbdullahTsai, Chia-LiangJaradat, NidalTsai, Chia-WenJarajapu, YagnaTseng, Yi-TangJauregi, PaulaTsiani, EvangeliaJaworowska, AgnieszkaTsivgoulis, GeorgiosJayant, KumarTsofliou, FotiniJe, YoujinTsopmo, ApollinaireJeanes, YvonneTsoulfas, GeorgeJeckel, Kimberly MTsuduki, TsuyoshiJeffery, Elizabeth HTu, ThomasJelski, WojciechTuck, CarolineJena, Prasant KumarTucker, Jared M.Jenkins, DavidTucker, Larry A.Jensen, Megan E.Tucker, RobinJeong, Seon-YongTueros, ItziarJeppesen, Per BendixTufarelli, VincenzoJericho, HilaryTullai, JohnJerums, GeorgeTume, LyvonneJeżewska-Zychowicz, MarzenaTur, JosepJiang, BaishanTuriel, MaurizioJiang, ChengTurner, JustineJiao, LiTurner, MarkJimenez-Chillaron, JosepTurpin-Nolan, SarahJimenez-Rondan, FelixTurrini, EleonoraJin, MirimTuuri, GeorgiannaJohnson, IanTuvdendorj, DemidmaaJohnson, LauraTwigger, Alecia-JaneJohnson, Sarah A.Tyson, Crystal CenellJohnson, TriciaUberti, DanielaJohnston, CarolUberti, FrancescaJohnston, Kelly L.Ubhayasekera, Sarojini J. K. A.Jones, JulieUebanso, TakashiJones, KerryUehara, MarikoJones, MarjorieUeno, Shu-ichiJongbloed, FrannyUlisse, SalvatoreJoo, Nam-seokUllrich, ReinerJorda, Marta AlegretUlrich, CraigJørgensen, Anders PalmstrømUmano, Giuseppina RosariaJoris, Peter J.Umar, Akhilesh KJoseph, BealsUnchwaniwala, NuruddinJospe, MichelleUnno, KeikoJówko, EwaUpadhyay, AbhinavJoyce, BrianUpadhyaya, JasbirJu, XiangqunUribarri, JaimeJuan, WenyenUsai Satta, PaoloJudge, Lawrence WUusi-Rasi, KirstiJuge, NathalieUusitupa, MattiJukić Peladić, NikolinaVaarno, JenniJulia, ChantalVaccaro, OlgaJulius, UlrichVachali, PreejithJun, Jonathan CVafeiadi, MarinaJung, SeungyounVafiadaki, ElizabethJuntti-Berggren, LisaVaidya, AmitaJuonala, MarkusVaidyanathan, BharatJurewitsch, BrianVaillancourt, CathyJyväkorpi, Satu KValdés-Ramos, RoxanaK A Foyez, MahmudVale, PauloK Song, MoonValencak, TeresaKabisch, StefanValenti, PieraKadioglu, OnatValentini, LuziaKagawa, MasaharuValentovic, MonicaKähkönen, Mika A.Valenzuela, RodrigoKahleova, HanaValera-Gran, DesiréeKaido, ToshimiValitutti, FrancescoKaklamanou, DaphneVamanu, EmanuelKalanetra, KarenVan Amburgh, MichaelKalhan, SatishVan Ballegooijen, Adriana JKaliora, Andriana C.Van Ballegooijen, HanneKalkman, Hans OVan Breda, SimoneKalliopi, KotsaVan Buiten, CharleneKamada, NobuhikoVan De Heijning, Bert J. M.Kamaly, NazilaVan Der Haar, FritsKamenov, KaloyanVan Der Laarse, Willem J.Kamiloglu, SenemVan Herck, Mikhaïl A.Kamolz, LarsVan Loon, LucKampmann, UllaVan Rossem, LenieKanakry, Christopher G.Van Schothorst, EvertKandala, Ngianga-BakwinVan Wymelbeke, VirginieKanekiyo, TakahisaVance, TerrenceKang, HyojeungVandenplas, YvanKang, InhaeVander Wal, JillonKang, Jae SeungVanderhoof, Jon A.Kang, SinyoungVanDusseldorp, TrishaKang, Sun ChulVapaatalo, HeikkiKang, Young-HeeVarady, KristaKannan, MuthukumarVarela-López, AlfonsoKanno, YoshihikoVargas, Jorge H.Kanojia, DeepakVarikuti, SanjayKantono, KevinVasilevsky, NicoleKao, Yung-HsiVásquez, ElizabethKaplan, Bonnie J.Vatner, Daniel FKappus, RebeccaVaughan, Roger A.Karaki, Shin-ichiroVaz, JosianaKarakochuk, CrystalVeeramani, SureshKaraman, SinemVejux, AnneKarim, Mohammad RezaulVeleeparambil, ManojKarim, NasiaraVenalainen, TaisaKarkhanis, VrajeshVenkatesh, SiddarthKarlsson, ErikVenn, BernardKarras, SpyridonVenskutonis, Petras RimantasKasarda, DonaldVentrella, DomenicoKaškonienė, VilmaVepsäläinen, HennaKass, Lindsy S.Verd, SergioKatada, ShunVergara, DanieleKatayama, ShigeruVerma, AnilKato, HiroyukiVerma, Madan LalKatsila, TheodoraVéronique, Demers-MathieuKattelmann, KendraVerpeut, JessicaKatuchova, JanaVerri, TizianoKatz, YitzhakVersino, ElisabettaKatzmarzyk, PTVeskoukis, Aristidis S.Kaur, GunveenVetter, Stefan W.Kaur, SimranjeetVicente-Salar, NéstorKawai, ShinichiViennois, EmilieKawai, YoshichikaVilaro, Melissa J.Kawamura, TomoyukiVilela, SofiaKawano, KouichiroVilla-Bellosta, RicardoKawasaki, KiyonoriVillalonga, PriamKay, Melissa C.Villano, DeboraKaye, ElizabethVillares, Jose Manuel MorenoKeane, DavidVillarini, AnnaKearns, CristinVillers, AgnèsKeat Wei, LooVincent, GraceKeating, ElisaVinceti, MarcoKeenan, MichaelVinciguerra, ManlioKeijer, JaapVinogradov, EvgueniiKeller, AmélieVinson, JoeKeller, HeatherVioque, JesusKelley, EricVirginio Agostoni, CarloKelly, Owen J.Visioli, FrancescoKennedy, David O.Visser, AnitaKennel, JulieVitale, MarilenaKenntner- Mabiala, RamonaVitetta, LuisKent, KatherineVitoria Miñana, IsidroKepczynska, MalgorzataVlassopoulos, AntonisKerhervé, HugoVogt, Leonie M.Kerr, DeborahVolicer, LadislavKerr, JessicaVollmer, LoriKeshavarzian, AliVolpe, StellaKeshteli, Ammar HassanzadehVon Hurst, P.Keyserling, ThomasVon Schacky, ClemensKhachik, FredVonk, RoelKhadka, ManojVoortman, TrudyKhan, AkbarVork, LisaKhan, Saira A.Vormann, JürgenKhatri, BhuwanVoziyan, PaulKhatri, KshitijVozza, IoleKhosla, PramodVucenik, IvanaKiełczykowska, MałgorzataWaalen, JillKieliszek, MarekWada, JunKiely, MaireadWada, YasuakiKienesberger, PetraWaddell, JaylynKiesswetter, EvaWadsack, ChristianKiewiet, M. B. G.Wagner, AnikaKilari, SreenivasuluWagner, CarolKim, Jae KyeomWaldmeier, FelixKim, JaehanWalker, MarjorieKim, Ji YeonWallace, AlisonKim, Ju-YoungWallace, AngelaKim, Na-HyungWallace, DanielKim, Ok-SuWallace, NicholasKim, SoJungWallace, RuthKim, YanghaWallis, GarethKim, YeonsooWalsh, AdamKim, YuriWalton, Gemma EmilyKimball, Samantha M.Walyaro, DJKing, JanetWang, Chin-KunKinsky, Suzanne MWang, DantongKipp, Anna PatriciaWang, DongKippler, MariaWang, Fan-FenKirk, BenWang, Feng-ShengKiss, Robert S.Wang, KaiKitson, Matthew T.Wang, KimberleyKitts, DavidWang, PengchengKlaus, SusanneWang, QiangKleiner, SusanWang, ShengnianKlotz, ChristianWang, XiaowanKnaapila, AnttiWang, YifeiKneepkens, C. M. FrankWang, ZenengKnight, KathyWangchuk, PhurpaKnight-Agarwal, CathyWard, LeighKnott, RachelWardenaar, FlorisKnudsen, Knud Erik BachWarshawsky, Bryna F.Knupp, GerdWatanabe, MikikoKnutsen, SveinWaterman, CarrieKoch, StefanWatkins, Adam J.Koch, WojciechWatkins, JuliaKocot, JoannaWatson, Emily J.Kodera, YukihioroWatt, PeterKoenig, Mary DawnWawer, AnnaKoenigstorfer, JoergWebb, KarenKoga, Jun-ichiroWeber, DanielaKoh, Gar YeeWeber, JanetKoh, Ho-JinWei, ChangyongKohan, RollandWei, ZhangKohler, Lindsay N.Weidner, SteffenKok, DieuwertjeWeller, RichardKok, WouterWelsh, Jean A.Kolarik, Andrew J.Wen, JianjunKoneru, BhuvaneswariWen, XiaominKones, RichardWen, XiaoyanKonić- Ristić, AleksandraWendell, Stacy GelhausKönig, DanielWessels, IngaKontogiorgis, ChristosWesson, DonKoppe, Janna G.West, DanielKoppe, LaetitiaWest, KeithKoppelman, Stef J.Westerterp-Plantenga, MargrietKornhaber, RachelWeyermann, MariaKornprat, PeterWeylandt, Karsten H.Korre, MariaWham, CarolKorte, CassandraWhelan, JillKorzeniowska, MargorzataWhisner, CorrieKoshinaka, KeiichiWhisner, Corrie M.Koskela, AliWhite, David J.Koulman, AlbertWhite, Robin E.Koutelidakis, Antonios E.Whitelock, VictoriaKovatcheva-Datchary, PetiaWibowo, DavidKowal, MałgorzataWichers, HarryKowalczewski, PrzemysławWiesner, JacquelineKoyama, YuWiesner-Reinhold, MelanieKrähenbühl, StephanWiggs, MichaelKrajewska-Włodarczyk, MagdalenaWightman, Emma L.Krall, Jodi S.Wigle, JeffreyKrämer, IreneWijayatunga, NadeejaKrawczyk-Lipiec, JoannaWilkinson, J. M.Krawinkel, MichaelWilkinson, Tom J.Kreider, RichardWillems, MarkKretschmer, NadineWilliams, AlisonKrieger, Jean-PhilippeWilliford, HankKrishna, SmritiWilson, EricaKrishnareddy, SuneetaWilson, PatrickKroeger, Cynthia M.Wilson, TedKroeze, WillemiekeWinek, KatarzynaKroon, PaulWinham, DonnaKruger, MarlenaWin-Shwe, Tin-TinKrupa-Kozak, UrszulaWirth, MichaelKrupp, DanikaWitard, OliverKruseman, MaaikeWithrow, NicoleKruzel, Marian L.Witkowska, Anna MariaKrzysztof Blusztajn, JanWłodarczyk, MartaKu, SeockmoWłodarek, DariuszKuan, Yu-HsiangWnuk, Susan MargaretKubachka, KevinWolber, FranKubena, KarenWolfe, Robert R.Kubicova, LenkaWolnerhanssen, Bettina K.Kubis, Hans-PeterWölnerhanssen, Bettina KarinKuczmarski, MarieWolters, MaikeKujawska, MałgorzataWon, DoyeonKuk, Jennifer L.Wong, AndreaKukreja, SubhashWong, WilliamKukula-Koch, WirginiaWoodward, BillKulaga, ZbigniewWoodward, JeremyKumar, AshutoshWoting, AnniKumar, BinodWright, Atte VonKumar, Jothi DineshWright, MeleciaKumar, NitinWu, Chi-ReiKumar, PrakashWu, FeitongKundur, Avinash ReddyWu, HongliKuo, Ping-ChungWu, JasonKuo, ShIu-MingWu, Pei-FungKupchak, Brian R.Wu, Shu-JuKupczyński, RobertWu, Wen-TzuKuppusamy, ManiselvanWulaningsih, WahyuKuppuswamy, AnnapoornaWulff, Anders BergKurdowska, Anna K.Wullaert, AndyKurien, Biji TheyilamannilWunderlich, Shahla M.Kuroda, KeijiWurtman, RichardKuwabara, MasanariWydau, SandraKuwahara, KeisukeXepapadaki, ParaskeviKwan, Sze Ting (Cecilia)Xiang, DongxiKwon, HyokjoonXiao, LiKwon, OranXiaoli, AlusKwong, Waldan K.Xinyin, JiangKyriazis, George A.Xu, Feng-LianKyrozis, AndreasXu, FurongL’Abbe, MaryXu, JingLa Rocca, Renato V.Xu, SuowenLa Vecchia, CarloXu, XiangruLa Vieille, SébastienXue, XiangLaatikainen, ReijoYada, ToshihikoLabruna, GiuseppeYamada, SatoruLachat, CarlYamada, SohsukeLachenmeier, DirkYamada, SonokoLagouri, VasilikiYamada, YosukeLaguna, Joan-CarlesYan, ShengminLaguzzi, FedericaYanda, MuraliLahiri, AmitYang, Chung-shuLahooti, HooshangYang, Jae JeongLai, Ching TatYang, JianLai, HeidiYang, KaiyuanLai, I-JuYang, QiyuanLai, YandongYang, Wai YewLaird, EamonYang, Wei-hsiungLalor, PatriciaYang, XiaoLamacchia, CarmelaYang, YingLambrinou, Christina P.Yang, YongkangLamport, Daniel J.Yang, YueLan, AnnaïgYano, AkiraLandrier, Jean-FrançoisYano, ShozoLang, ChimYao, JiayiLanigan, JulieYao, YuchengLanou, Amy JoyYasuda, KazukiLansdown, AlanYatabe, JunichiLanza, GiuseppeYazbeck, RogerLapid, KfirYe, JiangchuanLarsen, JunillaYeh, Shu-LanLasekan, John B.Yeo, SynLass, AchimYeomans, MartinLassalle, CamilleYi, TaoLatunde-Dada, Gladys OluyemisiYilmaz, BahtiyarLau, Wei LingYin, CongcongLaughlin, Maren R.Yin, JieLauretani, FulvioYokota, TomoyaLaurikka, PilviYokoyama, YokoLauro, Maria RosariaYoneshiro, TakeshiLaursen, Martin FrederikYoshida, TadashiLaustsen, ChristofferYoshikawa, ToruLavender, AndrewYoshino, HironoriLaviano, AlessandroYou, HyunjuLaville, MartineYou, XiaonaLaville, MauriceYoung, MelissaLaw, CherryYoung, WayneLawrence, KarlYourkavitch, JenniferLayman, DonaldYousefi, PaulLazarus, JohnYu, MeifangLazzeri, ChiaraYu, Yan PingLe Huerou-Luron, IsabelleYu, ZhipingLe Ouay, BenjaminYuan, QiLê, Kim-AnneYuan, ZhihongLe, LapYusuf, KamranLea, Michael A.Zabetakis, IoannisLeach, Steven T.Zagarins, SofijaLecoultre, VirgileZaguła, GrzegorzLedeganck, Kristien J.Zahradka, PeterLee, Anne R.Zaid, HilalLee, Byong H.Zakrocka, IzabelaLee, Chien-HungZalewska, MagdalenaLee, Daniel T.Zamani, MaedehLee, ElaineZamora-Ros, RaulLee, Hwa JinZamyatnin, Jr., Andrey A.Lee, Joo HyoungZandonadi, Renata PuppinLee, Jung EunZang, LiqingLee, JunsooZanini, BarbaraLee, MyoungsookZanini, MilkoLee, Seung-YeonZaragoza Martí, AnaLee, Sun YoungZárate, RafaelLee, Tzong-ShyuanZarini, GustavoLee, Yen-HanZdzieblik, DeniseLeech, RebeccaZehbe, IngebergLefere, SanderZeman, Daniel MeierLegua, PilarZemel, MichaelLehmann, UndineZendo, TakeshiLehtisalo, JenniZeng, HuaweiLeibovitz, EyalZgaga, LinaLeikin-Frenkel, Alicia I.Zhang, DeliangLeimanis, MaraZhang, DuoLeiphrakpam, Premila DZhang, GuanshiLeis Trabazo, Maria RosauraZhang, JiLeiva-Brondo, MiguelZhang, JianLems, WillemZhang, QianLengyel, ImreZhang, WencaiLeone, SerenaZhang, YixuanLeonidas, Demetres D.Zhang, YuLeporatti, StefanoZhang, YuanLerchbaum, ElisabethZhang, YunkaiLeri, FrancescoZhang, ZhenbinLerin, CarlesZhang, ZhiyingLesgards, Jean-FrançoisZhao, NingningLessard, LauraZhao, QinLesser, LenardZhao, ZongminLevenson, CathyZheng, JoleneLevy, LouisZheng, Xin-QiangLevy, StevenZhou, JingyingLewinska, AnnaZhu, Fengqing MaggieLi, ChunyingZhu, QiuyuLi, DongmeiZiauddeen, NidaLi, DuoZiegler, EkhardLi, Jau-YiZilversmit Pao, LeahLi, LiZimmerman, EmilyLi, LianboZiouzenkova, OulianaLi, LixinZoidis, EvangelosLi, MilingZordi, Nicola DeLi, MingZotta, TeresaLi, RuiZou, ChunbinLi, ShiyouZou, LiangLi, ShuaiZou, PingLi, WeijuanZouboulis, ChristosLi, WeikaiZüllig, RichardLi, WenyiZuo, LiLi, XiaoguangZurita Ortega, FélixLi, Yan Chun


